# Acute Rheumatism and Sepsis

**Published:** 1903-06-27

**Authors:** 


### ACUTE RHEUMATISM AND SEPSIS.

That acute rheumatism is an infective disease there is no reasonable doubt, and that it is a microbic affection is almost conclusive, but that it is due to a specific micro-organism is still a matter of very considerable uncertainty. In moist and temperate climates the disease is endemic, but from time to time epidemics prevail in a manner similar to well-known microbic diseases, and, what is still more significant, it is also shown that the occurrence of rheumatic fever in epidemic form is most marked in damp autumnal seasons following a summer of drought. The many manifestations of rheumatism and the variety of the lesions, the oscillation of temperature, the sweating, present a striking resemblance to tuberculosis and pyæmia. In its tendency to recur and to be induced by exposure it resembles pneumonia and erysipelas, while in its tendency to relapse it resembles enteric fever, and to end spontaneously the exanthemata. Of the organisms most generally recognised as associated in some way with rheumatic fever the diplococcus of Poynton and Paine, the staphylococcus and the streptococcus pyogenes, are probably the best known. But that rheumatism is a disease due solely to any one of these, or to any other one organism, is absolutely inconclusive. Clinically it is commonly recognised, though seldom sufficiently emphasised, that a sore throat—infective follicular tonsillitis, or suppurative tonsillitis—is one of the commonest antecedents of the manifestations of acute rheumatism. These manifestations may be not only arthritis, endocarditis, pericarditis and pleurisy, but also chorea, erythema nodosum, and certain other erythematous eruptions, as well as cutaneous and subcutaneous effusions, including, not uncommonly, purpura, sometimes pneumonia, possibly idiopathic peritonitis, and certainly profound anæmia. Any of these conditions may follow septic sore throat. In puerperal sepsis the occurrence of simple arthritis, pleurisy, etc. suggests the close relationship between rheumatic fever and sepsis. Allowing, then, that septic processes may and do precede rheumatism almost certainly as causal factors, and that in the majority of cases the throat is the seat of these processes, we have an important indication for prophylaxis, viz. appropriate application of antiseptics, even in the slight sore throats of children.

